# NDV-D90 suppresses growth of gastric cancer and cancer-related vascularization

**DOI:** 10.18632/oncotarget.16563

**Published:** 2017-03-25

**Authors:** Hong Sui, Kaibing Wang, Rui Xie, Xi Li, Kunpeng Li, Yuxian Bai, Xishan Wang, Bin Bai, Dan Chen, Jiazhuang Li, Baozhong Shen

**Affiliations:** ^1^ Department of Medical Oncology, Harbin Medical University Cancer Hospital, Harbin 150040, China; ^2^ Department of Intervention, The Second Hospital Affiliated Harbin Medical University, Harbin 150086, China; ^3^ Division of Swine Disease, National Key Laboratory of Veterinary Biotechnology, Harbin Veterinary Medicine, Harbin 150069, China; ^4^ Department of Abdominal Surgery, Cancer Hospital, Chinese Academy of Medical Sciences and Peking Union Medical College, Beijing 100021, China; ^5^ Department of Radiology, The Fourth Hospital of Harbin Medical University, Harbin 150001, China

**Keywords:** gastric cancer, newcastle disease viruses (NDV), apoptosis, vascular endothelial growth factor A (VEGF-A), matrix metallopeptidase 2 (MMP-2)

## Abstract

Recent reports suggest promises on using oncolytic Newcastle disease viruses (NDV) to treat different cancers, while the effects of a NDV-D90 strain on gastric cancer remain unknown. Here we showed that NDV-D90 induced gastric cancer cell apoptosis in a dose-dependent manner in 3 gastric cancer cell lines BGC-823, SGC-7901 and MKN-28. Pronounced reduction in cell invasion was detected in NDV-D90-treated BGC-823 and SGC-7901 cells, but not in MKN-28 cells. The increases in cell apoptosis and reduction in cell growth in NDV-D90-treated gastric cancer cells seemingly resulted from augmentation of p38 signaling and suppression of ERK1/2 and Akt signaling. *In vivo*, orthotopic injection of NDV-D90 impaired tumor growth and induced intratumoral necrosis. Tumor cells that had been pre-treated with NDV-D90 showed defect in development of implanted tumor. Moreover, NDV-D90 appeared to reduce gastric tumor vascularization, possibly through suppression of vascular endothelial growth factor A and Matrix Metallopeptidase 2. Together, our data suggest that NDV-D90 may have potential anti-cancer effects on gastric cancer.

## INTRODUCTION

Gastric cancer is a prevalent cancer, in which malignant cells form in the lining of the stomach [1]. Nearly all gastric cancers are adenocarcinomas [2]. Although surgical removal of the part of stomach that bares tumor may be effective for early stage gastric cancer, the majority of gastric cancer patients are diagnosed at an advanced stage due to lack of early signs or symptoms of the disease [1]. Therefore, novel therapies are required for treatment of advanced gastric cancer that may be amendable by surgery.

The anti-cancer potential of Newcastle disease viruses (NDV) has been discovered since 1805s [3]. The selective targeting elimination of replicating tumor cells by NDV stem from the presence of defective Interferon signaling pathways in tumor cells, but an effective antiviral response to hamper viral replication in normal cells [3]. Recently, selection, modification and production of oncolytic NDV with minimal damage of the normal adjacent tissues have been achieved [4, 5]. Genetic editing of NDV to express immune modulators (i.e. GM-CSF) has amplified the effects of NDV on targeted cancer tissues, rendering them one of the most effective multi-target cancer vaccines [5]. Since the clinical study of oncolytic NDV, three strains of NDV, MTH-68 [6], NDV-HUJ [7] and PV701 [8–11] have now been used in phase I/II clinical trials for tumor treatment.

Very recently, a NDV-D90 strain that was isolated from natural sources in China, and was shown to specifically induce apoptosis of human lung adenocarcinoma cell line A549 through regulating caspases [12]. Moreover, application of NDV-D90 appeared to reduce tumor growth *in vivo* [13]. Furthermore, the anti-cancer effects of NDV-D90 were just reported in oral squamous cell carcinoma [14]. However, whether NDV-D90 may have similar effects on gastric cancer is unknown.

Here, we addressed this question. We found that NDV-D90 induced gastric cancer cell apoptosis in a dose-dependent manner in 3 gastric cancer cell lines BGC-823, SGC-7901 and MKN-28. Pronounced reduction in cell invasion was detected in NDV-D90-treated BGC-823 and SGC-7901 cells, but not in MKN-28 cells. The increases in cell apoptosis and reduction in cell growth in NDV-D90-treated gastric cancer cells seemingly resulted from augmentation of p38 signaling and suppression of ERK1/2 and Akt signaling. *In vivo*, orthotopic injection of NDV-D90 impaired tumor growth and induced intratumoral necrosis. Tumor cells that had been pre-treated with NDV-D90 showed defect in development of implanted tumor. Moreover, NDV-D90 appeared to reduce gastric tumor vascularization, possibly through suppression of vascular endothelial growth factor A (VEGF-A) and Matrix Metallopeptidase 2 (MMP-2). Together, our data suggest that NDV-D90 may have potential anti-cancer effects on gastric cancer.

## RESULTS

### NDV-D90 reduces gastric cancer cell growth *in vitro*

**Figure 1:**
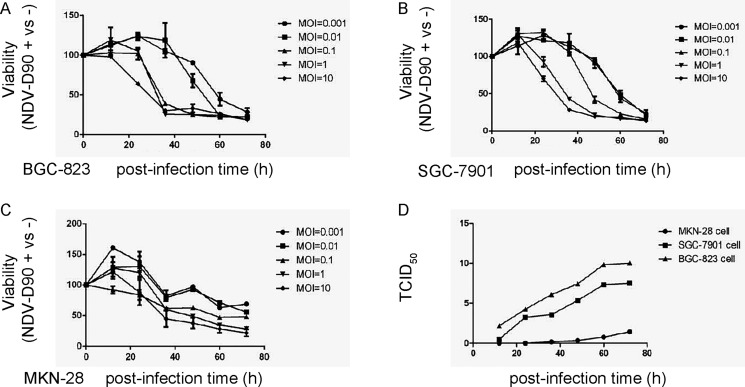
NDV-D90 reduces gastric cancer cell growth *in vitro* We treated 3 gastric cancer cell lines, BGC-823, SGC-7901 and MKN-28, with different MOI of NDV-D90, and analyzed cell viability at different time points in an CCK-8 assay. (**A**) CCK-8 assay for BGC-823. (**B**) CCK-8 assay for SGC-7901. (**C**) CCK-8 assay for MKN-28. (**D**) TCID_50_ for virus amplification in 3 cell lines. The amount of a pathogenic agent that will produce pathological change in 50% of cell cultures inoculated is expressed as TCID_50_/ml. *p < 0.05: BGC-823 vs MKN-28. ^&^*p* < 0.05: SGC-7901 vs MKN-28. *N* = 5.

First, we examined the effects of NDV-D90 on the growth of gastric cancer cells *in vitro*. We used 3 human gastric cancer cell lines, BGC-823, SGC-7901 and MKN-28. BGC-823 is low differentiated gastric carcinoma, SGC-7901 is medium differentiated gastric carcinoma, while MKN-28 is highly differentiated gastric carcinoma. We found that NDV-D90 dose-dependently reduced the cell viability in all 3 lines, and the effects of NDV-D90 on BGC-823 and SGC-7901 cells appeared to be more pronounced (Figure 1A–1C). Next, virus amplification was measured in the 3 cell lines, showing that viruses proliferated more quickly in BGC-823 and SGC-7901 cells, compared to MKN-28 cells (Figure 1D). Together, these data suggest that NDV-D90 reduces gastric cancer cell growth *in vitro*, and NDV-D90 may be more effective on low differentiated, highly proliferative gastric cancer cells.

### NDV-D90 reduces gastric cancer cell invasion *in vitro*

**Figure 2:**
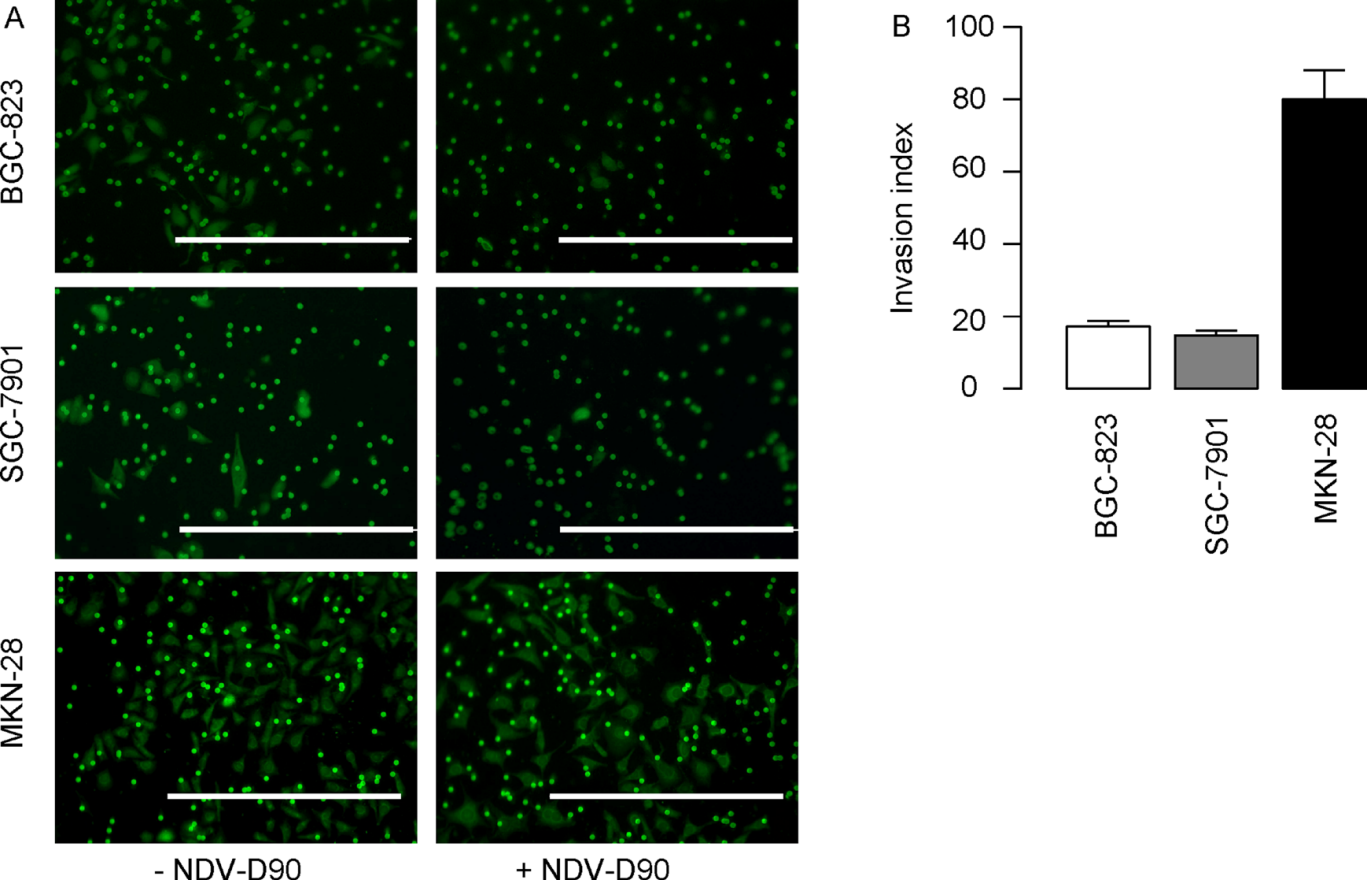
NDV-D90 reduces gastric cancer cell invasion *in vitro* Tumor cell invasion test was performed and the invasion index (here termed as the ratio of the NDV-D90-treated tumor cells that pass through the chamber versus the control tumor cells that pass through the chamber) was determined. (**A**) Representative images. (**B**) Quantification. Scale bars are 200 μm. *N* = 5.

We then examined the alteration of gastric cancer cell invasion after NDV-D90 treatment. Tumor cell invasion test was performed and the invasion index (here termed as the ratio of the NDV-D90-treated tumor cells that pass through the chamber versus the control tumor cells that pass through the chamber) was determined. We found that NDV-D90 treatment reduced the invasion index by 83% in BGC-823 cells, by 85% in SGC-7901 cells, but only by 20% in MKN-28 cells, shown by representative images (Figure 2A), and by quantification (Figure 2B). These data suggest that NDV-D90 reduces gastric cancer cell invasion *in vitro* and this effect may be more pronounced on low differentiated, highly proliferative gastric cancer cells.

### NDV-D90 induces gastric cancer cell apoptosis *in vitro*

**Figure 3:**
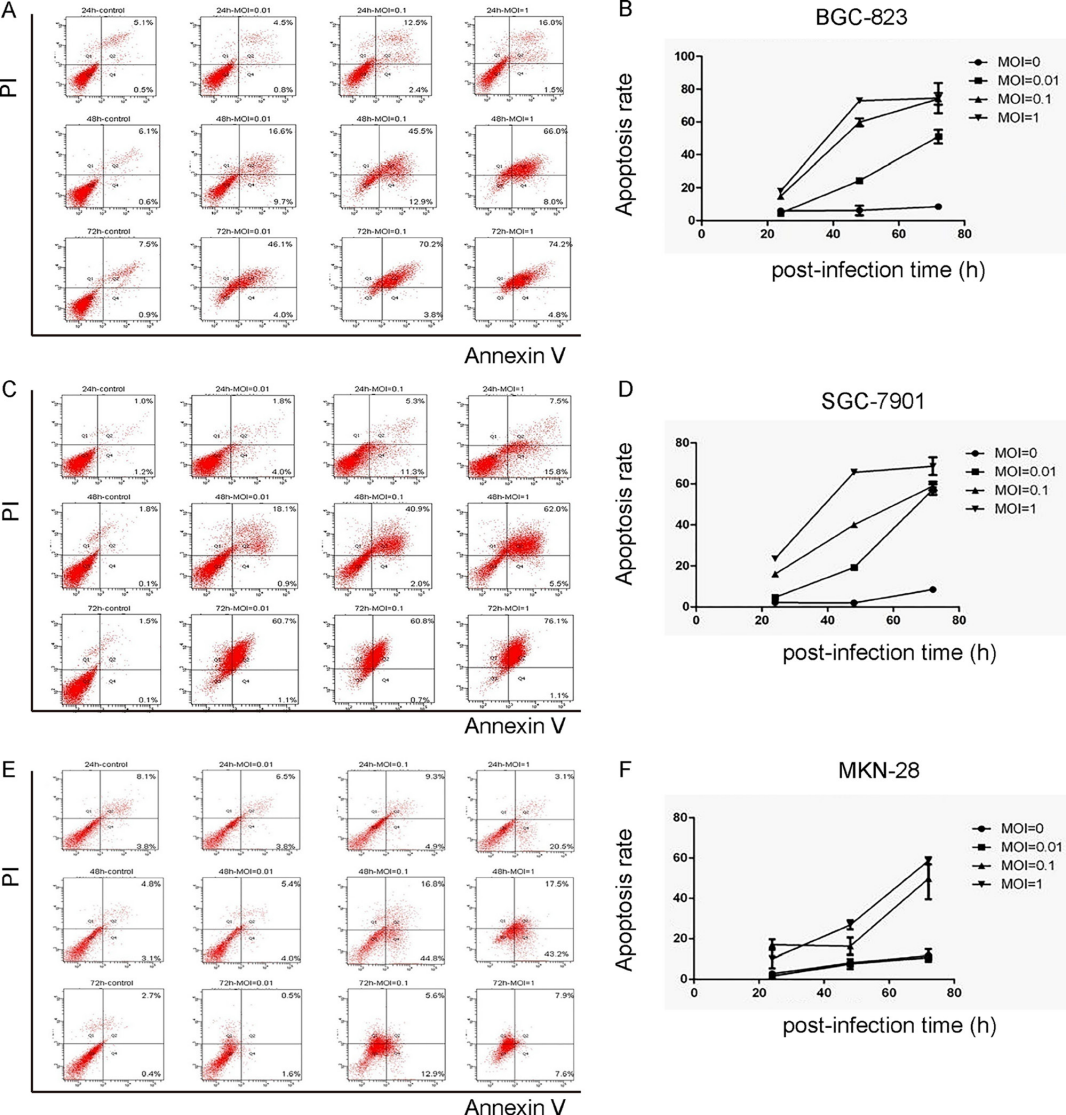
NDV-D90 induces gastric cancer cell apoptosis *in vitro* The effects of NDV-D90 on cell apoptosis were analyzed by Annexin V / PI assay using flow cytometry. (**A**) Representative flow charts for BGC-823. (**B**) Quantification for BGC-823. (**C**) Representative flow charts for SGC-7901. (**D**) Quantification for SGC-7901. (**E**) Representative flow charts for MKN-28. (**F**) Quantification for MKN-28. *N* = 5.

Next, we analyzed the effects of NDV-D90 on cell apoptosis. We found that NDV-D90 induced apoptosis in a dose-dependent manner in all 3 gastric cancer cell lines BGC-823 (Figure 3A–3B), SGC-7901 (Figure 3C–3D) and MKN-28 (Figure 3E–3F). Again, more pronounced reduction in cell invasion was detected in NDV-D90-treated BGC-823 and SGC-7901 cells, but not in MKN-28 cells (Figure 3A–3F).

### NDV-D90 enhances p38 signaling and inhibits ERK1/2 and Akt signaling in gastric cancer cells

**Figure 4:**
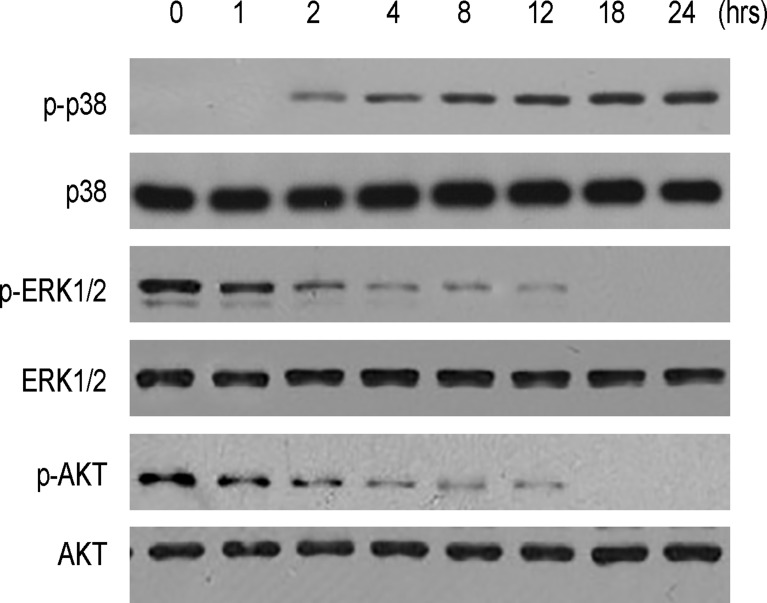
NDV-D90 enhances p38 signaling and inhibits ERK1/2 and Akt signaling in gastric cancer cells Representative Western blotting for phosphorylation of p38 (p-p38), p38, phosphorylation of ERK1/2 (p-ERK1/2), ERK1/2, phosphorylation of AKT and AKT after NDV-D90 treatment on BGC-823 cells. *N* = 5.

In order to understand the underlying mechanisms, we analyzed phosphorylation of a key factor in apoptosis-associated signaling pathway, p38, phosphorylation of 2 key factors in proliferation-associated signaling pathway, ERK1/2 and AKT. We found that NDV-D90 treatment induced the phosphorylation of p38, but suppressed the phosphorylation of ERK1/2 and AKT on BGC-823 cells (Figure 4). These data suggest that the increases in cell apoptosis and reduction in cell growth in NDV-D90-treated gastric cancer cells may result from augmentation of p38 signaling and suppression of ERK1/2 and Akt signaling.

### NDV-D90 reduces gastric cancer cell growth *in vivo*

The next question is whether NDV-D90 may have similar anti-cancer effects against gastric cancer *in vivo*. In order to examine the implanted tumor cells in living animals, we transduced

**Figure 5:**
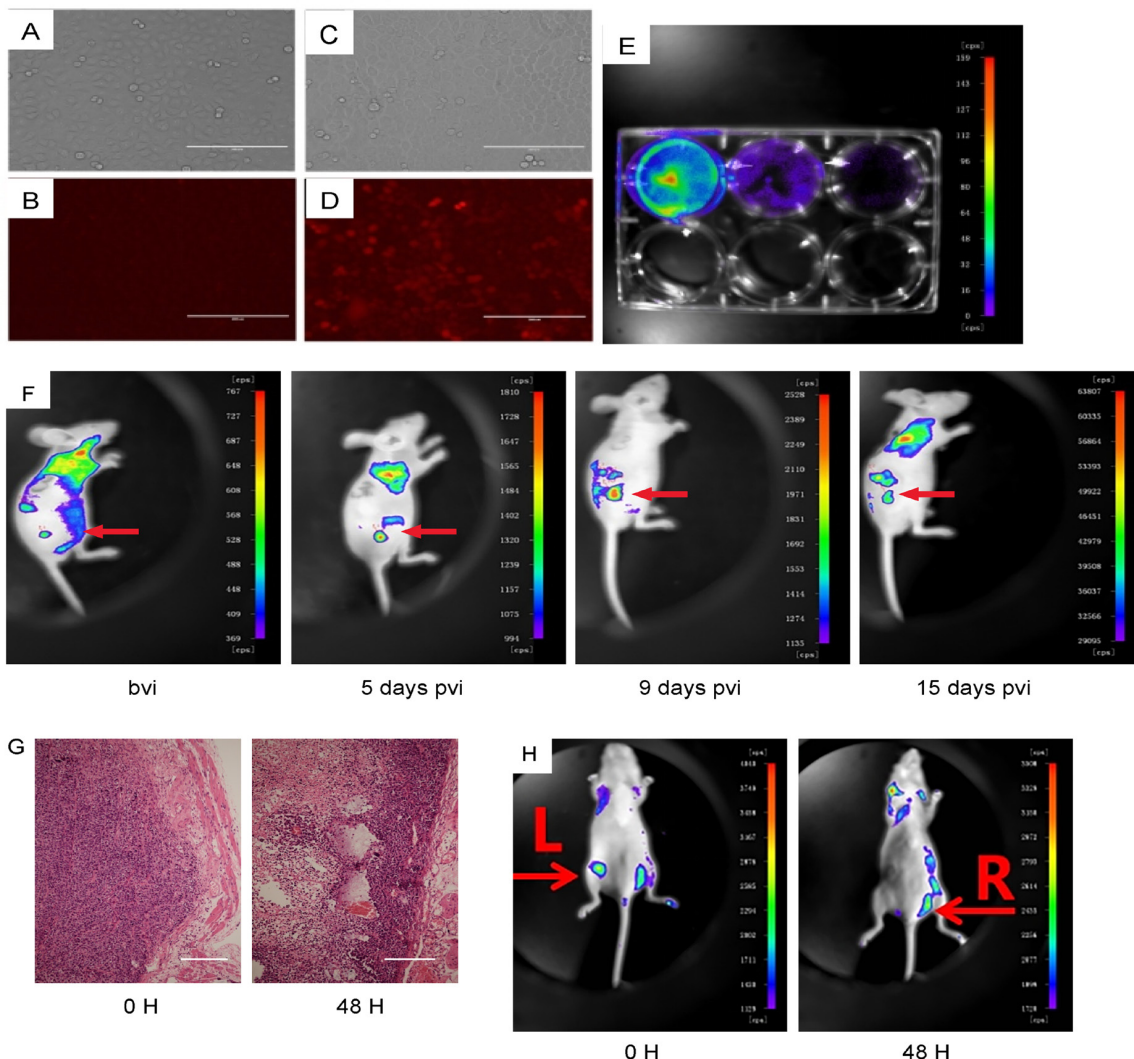
NDV-D90 reduces gastric cancer cell growth *in vivo* BGC-823 cells were transduced with lentivirus carrying a RFP reporter. (**A**) Bright field for non-transduced cells. (**B**) Red fluorescent field for non-transduced cells. (**C**) Bright field for transduced cells. (**D**) Red fluorescent field for transduced cells. (**E**) The transduced cells (termed as BGC-823-RFP) were validated under fluorescent microscopy *in vitro*. (**F**) BGC-823-RFP cells were then implanted into nude mice, after which NDV-D90 was intratumorally injected. The virally injected tumor (arrow) was monitored at different time points after viral injection, showing suppression of tumor growth by representative images. (**G**) Representative histological images showing presence of necrosis exclusively in the implanted BGC-823-RFP tumor treated with NDV-D90. (**H**) When BGC-823-RFP cells were pre-treated with NDV-D90 before implantation, the signals from the implanted tumor cells L: were hardly detected 48 hours after transplantation. R is control (BGC-823-RFP cells without NDV-D90 treatment). Bvi: before viral injection. Pvi: post viral injection. Scale bars are 100 μm. *N* = 5.

BGC-823 cells with lentivirus carrying a RFP reporter (Figure 5A–5D). The transduced cells (termed as BGC-823-RFP) were validated under fluorescent microscopy *in vitro* (Figure 5E). BGC-823-RFP cells were then implanted into nude mice, after which NDV-D90 was intratumorally injected. The tumor was monitored at different time points after viral injection, showing suppression of tumor growth by representative images (Figure 5F). Significant necrosis was detected exclusively in the implanted BGC-823-RFP tumor treated with NDV-D90 (Figure 5G). When BGC-823-RFP cells were pre-treated with NDV-D90 before implantation, we found that 48 hours after transplantation, the signals from the implanted tumor cells were hardly detected (Figure 5H). Together, these data support an anti-cancer role of NDV-D90 *in vivo* in gastric cancer.

### NDV-D90 impairs gastric cancer vascularization

**Figure 6:**
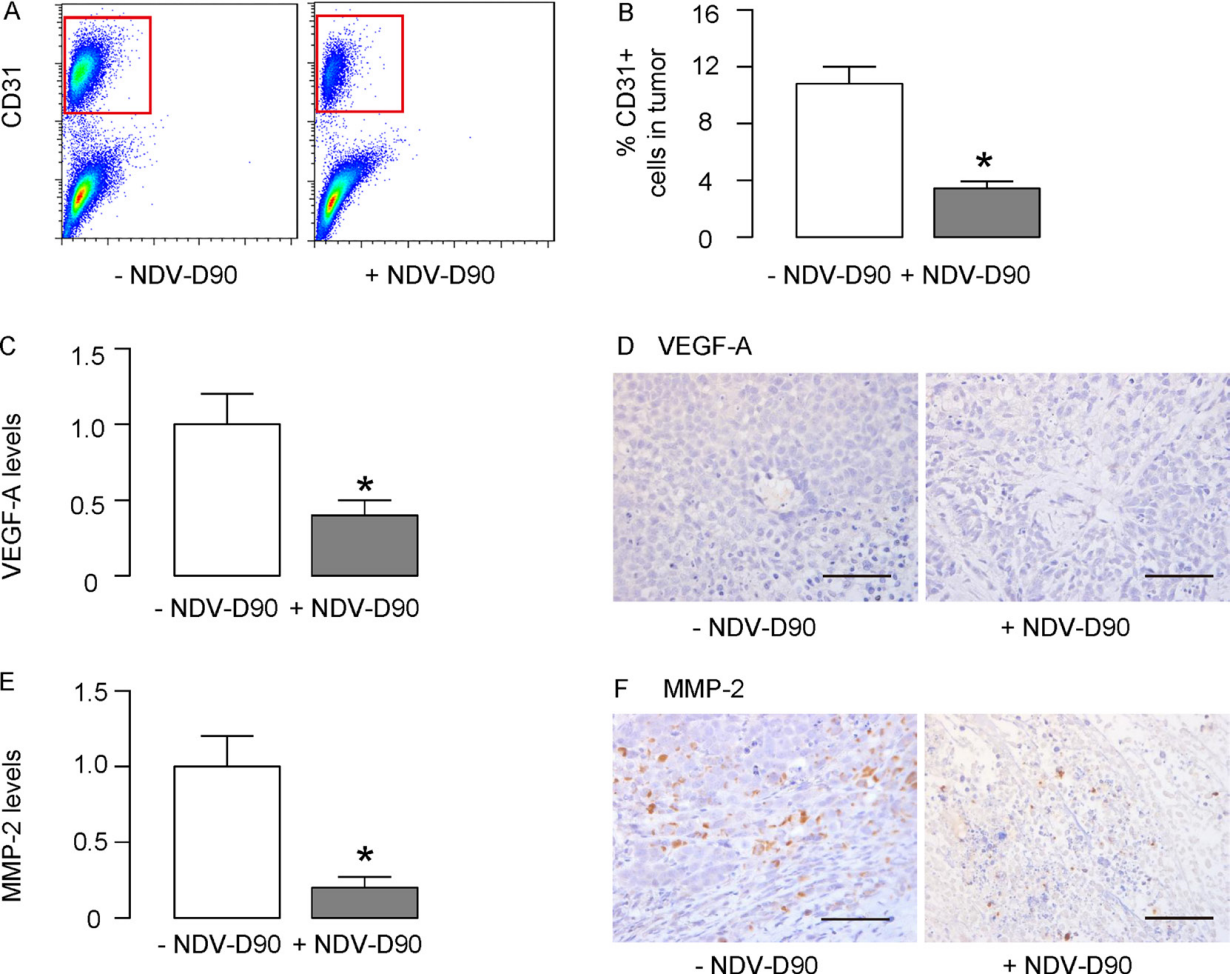
NDV-D90 impairs gastric cancer vascularization (**A**–**B**) Implanted tumors treated with/without NDV-D90 were dissociated and analyzed for the percentage of CD31+ endothelial cells inside the tumor, shown by representative flow charts (A), and by quantification (B). (**C**–**D**) ELISA (C) and immunohistochemistry (D) for VEGF-A in implanted tumor. (**E**–**F**) ELISA (E) and immunohistochemistry (F) for MMP-2 in implanted tumor.

Finally, we examined the effects of NDV-D90 on gastric cancer vascularization. Implanted tumors treated with/without NDV-D90 were dissociated and analyzed for the percentage of CD31+ endothelial cells inside the tumor. We found that NDV-D90 treatment significantly reduced the percentage of the CD31+ endothelial cells inside the tumor, shown by representative flow charts (Figure 6A), and by quantification (Figure 6B). We then examined the regulators of vascularization in the tumor and found that the levels of VEGF-A were significantly reduced in NDV-D90-treated tumor, compared to control, by ELISA (Figure 6C) and by immunohistochemistry (Figure 6D). Similarly, we found that the levels of MMP-2 were significantly reduced in NDV-D90-treated tumor, compared to control, by ELISA (Figure 6E) and by immunohistochemistry (Figure 6F). Thus, NDV-D90 appeared to reduce gastric tumor vascularization, possibly through suppression of VEGF-A and MMP-2. Together, our data suggest that NDV-D90 may have potential anti-cancer effects on gastric cancer.

## DISCUSSION

NDV is also known as avian paramyxovirus serotype 1, which belongs to a non-segmented, negative-strand RNA virus of the family Paramyxoviridae with a natural avian host range [3]. The genome of NDV is approximately 15 kb in length that encodes 6 structural proteins. Recent advance in application of virulence associated with human pathogens renders animal viruses as a promising therapeutic agent for tumor, since NDV does not have significant adverse effects in humans [3]. Interestingly, the selective targeting and the destruction of replicative tumor cells by NDV seemingly originates from the defective interferon signaling pathways in tumor cells, since interferon regulatory factor genes encode DNA-binding proteins that are involved in the innate immune response to viral infection [15].

Very recently, NDV-D90 strain was shown to induce apoptosis of human lung cancer cells and inhibit tumor growth *in vitro* [12], and *in vivo* [13]. In another independent study, the anti-cancer effects of NDV-D90 were detected in oral squamous cell carcinoma. However, whether NDV-D90 may have similar effects on gastric cancer is unknown. Moreover, the underlying mechanisms remain ill-defined. Here, we addressed these questions.

We found that NDV-D90 induced gastric cancer cell apoptosis and reduced cell invasion in a dose-dependent manner in 3 gastric cancer cell lines BGC-823, SGC-7901 and MKN-28. However, pronounced effects were detected in NDV-D90-treated BGC-823 and SGC-7901 cells, but not in MKN-28 cells. Since BGC-823, SGC-7901 and MKN-28 represent low differentiated, medium differentiated and highly differentiated gastric cancer cells respectively, these data suggest that NDV-D90 may be more effective on low differentiated, highly proliferative gastric cancer cells, which is supported by the high amplification of NDV-D90 in low differentiated, highly proliferative gastric cancer cells.

We detected suppression of VEGF-A and MMP-2 in NDV-D90-treated gastric cancer cells. Both VEGF-A and MMP-2 are associated with tumor-related vascularization [16–19]. Of note, MMP-2 was regulated by p38, ERK1/2 and Akt signaling pathway [20–22], while VEGF-A was similarly regulated by p38, ERK1/2 and Akt signaling pathway [23–25]. Therefore, although the increases in cell apoptosis and reduction in cell growth in NDV-D90-treated gastric cancer cells seemingly resulted from augmentation of apoptosis-associated p38 signaling and suppression of proliferation-associated ERK1/2 and Akt signaling, these signaling pathways also have effects on cell mobility and invasion potential.

Finally, we approved our *in vitro* findings *in vivo*. Orthotopic injection of NDV-D90 impaired tumor growth and induced intratumoral necrosis. The effects of NDV-D90 on tumor growth occurred earlier after treatment. In addition, the formation of the implanted tumor appeared to be affected by NDV-D90 treatment.

Although the current study investigated the effects of NDV-D90 on human gastric cancer cells, the tumor environment was in mice, but not in humans. The immunodeficiency state of nude mice may also have an effect on the interpretation of the data. Future studies may be performed in human patients to conquer these limitations. However, based on the collected evidence here, our data suggest that NDV-D90 may have potential anti-cancer effects on gastric cancer.

## MATERIALS AND METHODS

### Protocol approval

All the experimental methods in the current study has been approved by the research committee at Harbin Medical University. All the experiments have been carried out in accordance with the guidelines from the research committee at Harbin Medical University. All animal experiments were approved by the Institutional Animal Care and Use Committee at Harbin Medical University. Surgeries were performed in accordance with the Principles of Laboratory Care, supervised by a qualified veterinarian.

### Reagents and cell line culture

NDV-D90 was obtained from National Key Laboratory of Veterinary Biotechnology of Harbin Veterinary Medicine. Gastric cancer lines BGC-823, SGC-7901 and MKN-28 were purchased from Chinese Academy of Sciences Culture Collection (Shanghai, China), and maintained in RPMI 1640 medium (Hyclone, Shanghai, China) supplemented with 10% fetal bovine serum (Hyclone) in a humidified chamber with 5% CO_2_ at 37°C.

### Cell viability assay

The CCK-8 detection kit (DOJINDO, Shanghai, China) was used to measure cell viability. Briefly, gastric cancer cells were seeded in a 96-well microplate at a density of 10^4^/ml. After successful attachment, cells were treated with NDV-D90 at a multiplicities of infection (MOI) of 0.001, 0.01, 0.1 and 1, respectively, for a duration of 2 hours. The cells were cultured for 12 hours, 24 hours, 36 hours, 48 hours, 60 hours and 72 hours, after which 10 μl CCK-8 solution was added in each well and the plate was incubated at 37°C for 4 hours before absorbance was measured with a monochromator microplate reader at a wavelength of 450 nm. The optical density value was reported as the percentage of cell viability in relation to the control group (set as 100%).

### Measurement of virus amplification

Gastric cancer cells were seeded in a 6-well microplate at a density of 10^5^/ml. After successful attachment, cells were treated with NDV-D90 at a MOI of 0.01 for a duration of 2 hours. The cells were cultured for 12 hours, 24 hours, 36 hours, 48 hours, 60 hours and 72 hours, and the culture supernatant was collected at each time point for measurement of the TCID_50_.

### Tumor cell invasion test

Gastric cancer cells were seeded in a 6-well microplate at a density of 10^5^/ml. After successful attachment, cells were treated with NDV-D90 at a MOI of 0.01 for a duration of 2 hours. The infected cells were collected and 500 μl was added into the upper chamber of a BD BioCoat™ Matrigel™ Invasion Chamber (Becton-Dickinson Biosciences, San Jose, CA, USA) at a density of 5 × 10^4^/ml. The lower chamber was added with 750 μl culture medium containing 10% FBS. The incubation time was 14 hours, after which 50 μl Calcein-AM staining solution was added for 30 minutes to allow cells to be stained and visualized by fluorescent microscopy.

### Apoptosis assay and flow cytometry

Cells were labeled with annexin V-FITC and propidium iodide (PI), using an apoptosis detecting kit (KeyGEN Biotech, Nanjing, China), and analyzed by flow cytometry using CellQuest software (Becton-Dickinson Biosciences, San Jose, CA, USA). For analyzing CD31+ cells in implanted tumor, the tumor was resected, minced into small pieces, and then digested in the digestion media containing 40 mg/dl collagenase (Sigma-aldrich, San Jose, CA, USA) and 0.05% trypsine (Sigma-Aldrich) at 37°C for 30 min. After the digestion, the cells that passed a 40 μm filter were subjected to flow cytometric analysis and sorting, using a FITC-conjugated rat-anti-mouse CD31 antibodiy (Becton-Dickinson Biosciences). Data were analyzed using FlowJo software (Flowjo LLC, Ashland, OR, USA).

### Western blot

Protein was extracted from the cultured cells with RIPA lysis buffer (1% NP40, 0.1% Sodium dodecyl sulfate (SDS), 100 μg/ml phenylmethylsulfonyl fluoride, 0.5% sodium deoxycholate, in PBS) on ice. Protein concentration was determined using a BCA protein assay kit (Bio-rad, China), and whole lysates mixed with 4×SDS loading buffer (Bio-rad, China) were denatured by 5 minutes’ incubation at 100°C for 5 min. Afterwards, proteins were separated on SDS-polyacrylamide gels, and then transferred to a PVDF membrane. The membrane blots were first probed with a primary antibody. After incubation with horseradish peroxidase-conjugated second antibody, protein was visualized using an enhanced chemiluminescent system. Primary antibodies were rabbit anti-phosphorylated p38 (p-p38), anti-p38, anti-phosphorylated ERK1/2 (p-ERK1/2), anti-ERK1/2, anti-phosphorylated AKT (p-AKT) and anti-AKT (all purchased from Cell Signaling, San Jose, CA, USA). Secondary antibody is HRP-conjugated anti-rabbit (Jackson ImmunoResearch Labs, West Grove, PA, USA).

### ELISA

ELISA was performed using mouse VEGF-A or MMP-2 ELISA kit (R&D System, Los Angeles, CA, USA) according to manufacturer's instruction.

### Immunohistochemistry

Immunohistochemistry was performed using a HRP/DAB (ABC) Detection IHC kit (Abcam, Cambridge, MA, USA) according to manufacturer's instruction. Both rabbit anti-VEGF-A and anti-MMP2 were purchased from R&D System.

### Nude mouse tumor model

BGC-823 cells were transduced with lenti-RFP viruses (ABM, Shanghai, China) to allow visualization of them in the implanted tumor by fluorescent microscopy. The male nude mice were purchased from SLAC Laboratory Animal Co. Ltd (Shanghai, China). These mice were used at 12 weeks of age, when they received subcutaneous transplantation with 108 gastric cancer cells on the back. After 3 weeks, the formed tumor received intratumoral injection of NDV-D90 or PBS as a control.

### Statistical analysis

All statistical analyses were carried out using the SPSS 18.0 statistical software package. All data were statistically analyzed using one-way ANOVA with a Bonferroni correction, followed by Fisher's exact test to compare two groups. All values in cell and animal studies are depicted as mean ± standard deviation and are considered significant if *p* < 0.05.
